# Comparative efficacy and safety of apixaban and rivaroxaban versus warfarin in atrial fibrillation patients with end-stage renal disease: a systematic review and meta-analysis

**DOI:** 10.1080/0886022X.2026.2666454

**Published:** 2026-05-13

**Authors:** Xiaoyuan Wu, Jie Meng, Yu Yang, Dacheng Sheng, Xin Liu, Yilun Zhou

**Affiliations:** a Department of Nephrology, Beijing Tiantan Hospital, Capital Medical University, Beijing, China; b Department of Pathology, Beijing Tongren Hospital, Capital Medical University, Beijing, China; c Department of Cardiology, Beijing Tiantan Hospital, Capital Medical University, Beijing, China; d Department of Pharmacy, Beijing Tiantan Hospital, Capital Medical University, Beijing, China

**Keywords:** Apixaban, rivaroxaban, atrial fibrillation, end-stage renal disease, dialysis

## Abstract

This study aimed to evaluate the comparative efficacy and safety of apixaban and rivaroxaban versus vitamin K antagonists (VKAs) in anticoagulation management in a dialysis population. PubMed, Embase, and the Cochrane Library were searched for studies comparing apixaban or rivaroxaban with VKAs in patients with atrial fibrillation (AF) undergoing dialysis. The primary efficacy endpoints included stroke/systemic embolism (SSE) and all-cause mortality. Safety outcomes encompassed major bleeding, intracranial hemorrhage, and gastrointestinal bleeding. Risk ratios (RR) with 95% confidence intervals (CI) were synthesized using random-effects models. The meta-analysis included three randomized controlled trials (RCTs) and eight observational studies. Pooled analyses showed that apixaban and rivaroxaban were associated with lower risks of major bleeding (RR 0.57, 95% CI: 0.51–0.63), gastrointestinal bleeding (RR 0.66, 95% CI: 0.57–0.76), and intracranial hemorrhage (RR 0.54, 95% CI: 0.36–0.83) compared with VKAs. Additionally, apixaban and rivaroxaban were associated with reduced risk of SSE (RR 0.57, 95% CI: 0.46–0.72) and all-cause mortality (RR 0.73, 95% CI:0.63–0.83), although substantial heterogeneity was present. Exploratory dose-stratified analyses suggested both standard- and low-dose apixaban regimens were associated with favorable efficacy and hemostatic safety relative to warfarin. Consistent numerical trends were observed in the RCT-only analysis, though none reached statistical significance owing to limited sample size. In conclusion, apixaban and rivaroxaban are associated with lower risks of bleeding compared with VKAs in patients with AF and ESRD. However, evidence regarding their efficacy in preventing SSE, all-cause mortality and the optimal apixaban dosing regimen remains inconclusive and requires validation in large, dedicated RCTs.

## Introduction

Atrial fibrillation (AF) affects 13–27% of patients with end-stage renal disease (ESRD) undergoing hemodialysis, a prevalence markedly higher than that in the general population [1,2]. Dialysis-related triggers, such as acute electrolyte shifts, hemodynamic instability (e.g. rapid blood volume or pressure fluctuations), myocardial stunning, and autonomic dysfunction, contribute to AF pathogenesis in this population [3,4]. AF increases the thromboembolic risk by a factor of 4–5, with ischemic stroke being the predominant complication [5]. Although oral anticoagulation effectively reduces stroke incidence, its use in the dialysis population requires careful benefit–risk evaluation owing to the concurrent high risks of thrombosis and hemorrhage [6].

In recent years, a non-pharmacological option, left atrial appendage closure, has been employed to prevent AF-associated stroke, particularly in dialysis patients [7]; however, pharmacological therapy remains the mainstream approach. Direct oral anticoagulants (DOACs), particularly apixaban and rivaroxaban, have emerged as alternatives to vitamin K antagonists (VKAs) owing to fixed dosing, the absence of routine monitoring, and fewer drug–food interactions. Although warfarin remains the primary anticoagulant for patients with ESRD, its narrow therapeutic window, international normalized ratio (INR) variability, and associations with accelerated nephropathy and vascular calcification limit its long-term use [8–10]. However, the safety and efficacy of apixaban and rivaroxaban in patients undergoing dialysis remain unclear. A meta-analysis including three randomized controlled trials (RCTs) comparing apixaban or rivaroxaban with VKAs failed to demonstrate significant differences in outcomes, likely because of underpowered sample sizes [11]. Additionally, large-scale retrospective cohort studies and database analyses have yielded conflicting conclusions, and apixaban dosing remains particularly contentious. While some studies advocate standard dosing (5 mg twice daily) [12,13], others support a lower dose (2.5 mg twice daily) [14–16], with additional evidence suggesting comparable outcomes across dosing regimens.

Given the persistent clinical equipoise and paucity of robust randomized evidence in dialysis-dependent populations, we conducted a comprehensive meta-analysis integrating both RCTs and observational studies to evaluate the comparative efficacy and safety of apixaban and rivaroxaban versus VKAs in patients with ESRD undergoing maintenance dialysis where anticoagulation was clinically indicated after shared decision-making.

This meta-analysis offers three key contributions that add value beyond prior systematic reviews. First, it incorporates newly available evidence published through May 2024, including studies by Moore et al. and Laville et al. that were absent from earlier syntheses. Second, it focuses exclusively on apixaban and rivaroxaban, excluding dabigatran (which is not approved by the Food and Drug Administration (FDA) for dialysis patients and is predominantly renally excreted), thereby minimizing pharmacological heterogeneity. Third, unlike previous analyses that pooled mixed populations (e.g. AF and venous thromboembolism), our study targets dialysis-dependent patients with AF and includes dose-stratified comparisons of standard (5 mg twice daily) and reduced (2.5 mg twice daily) apixaban regimens to address the unresolved dosing controversy and inform optimal anticoagulation strategies in this vulnerable population. A detailed comparison with the most relevant recent meta-analyses is provided in Supplementary Table S1, summarizing differences in search end dates, study eligibility criteria, anticoagulant scope, dose-specific analyses, and outcome reporting.

## Methods

This meta-analysis was conducted in accordance with the guidance provided in the Cochrane Handbook for Systematic Reviews. Two reviewers (X.W. and D.S.) independently performed the literature search, study selection, data abstraction, quality assessment, and data analysis. Disagreements were resolved by discussion between the two reviewers or consultation with the corresponding authors. The protocol of this study was registered on the PROSPERO platform (CRD42025635660). Minor deviations from the original protocol were made during the conduct of the review and are documented in Table S2 of the Supplementary Materials. The PRISMA 2020 Checklist for the present meta-analysis is presented in Supplementary Files (Supplementary Table S3).

### Inclusion and exclusion criteria

Eligible RCTs and observational cohort studies were included if they compared at least one efficacy or safety outcome of factor Xa inhibitors (apixaban or rivaroxaban) versus warfarin in AF patients with ESRD on dialysis (hemodialysis or peritoneal dialysis), where anticoagulation was clinically indicated after shared decision-making. Efficacy outcomes included the composite of stroke/systemic embolism (SSE) and all-cause mortality. The safety outcomes were major bleeding, intracranial bleeding, and gastrointestinal bleeding. Outcome definitions were adopted and reported in the original studies. Studies were excluded if they involved patients with AF undergoing cardioversion, ablation, or left atrial appendage occlusion. Other article types (e.g. reviews, comments, case reports, case series, letters, editorials, and meeting abstracts) were also excluded.

### Literature search

PubMed, Embase, and the Cochrane Library were systematically searched from inception to May 7, 2024, to identify studies on the effectiveness and safety of factor Xa inhibitors (apixaban and rivaroxaban) compared with warfarin in patients with AF and ESRD on dialysis. The following searching items were combined using “AND” [1]: “atrial fibrillation,” [2] “dialysis” OR “hemodialysis” OR “peritoneal dialysis” OR “end-stage kidney disease” OR “end-stage renal disease” OR “advanced renal disease,” [3] “vitamin K antagonist” OR “warfarin” [4] “non-vitamin K antagonist oral anticoagulant” OR “direct oral anticoagulant” OR “novel oral anticoagulant” OR “NOAC” OR “DOAC” OR “factor Xa inhibitors” OR “apixaban” OR “rivaroxaban.” No language restrictions were applied.

### Study screening and data abstraction

We first screened the titles and abstracts of all retrieved studies and then reviewed the full texts of potential studies. Final inclusion was based on the pre-defined inclusion criteria. The following data were extracted from each included study: first author, publication year, study design, patient characteristics (e.g. study population, sample size, age, and sex), type and dosage of DOACs, duration of follow-up, and the effectiveness and safety outcomes.

### Study quality assessment

Eligible RCTs were independently assessed by two researchers for risk of bias using the Cochrane Collaboration’s criteria [17]. The assessment covered five items [1]: bias arising from the randomization process [2], bias due to deviations from intended interventions [3], bias due to missing outcome data [4], bias in measurement of the outcome, and [5] bias in selection of the reported result. Risk of bias was graded A (low risk), B (unclear), or C (high risk). For observational studies, the “Risk of Bias in Non-randomized Studies-of Interventions” (ROBINS-I) tool was applied to each study [18]. The ROBINS-I tool addresses the following domains [1]: bias due to confounding [2], bias in selection of participants into the study [3], bias in classification of interventions [4], bias due to deviations from intended interventions [5], bias due to missing data [6], bias in measurement of outcomes, and [7] bias in selection of the reported result.

### Quantitative data synthesis

Meta-analyses were conducted separately for each outcome. To evaluate the robustness and consistency of findings, we performed three distinct sets of analyses [1]: a crude-event-based analysis, in which studies reporting raw event counts were included, and risk ratios (RRs) with 95% confidence intervals (CIs) were pooled using the Mantel–Haenszel method under a random-effects model, this approach constitutes the primary analysis of our study [2]; an adjusted-effect analysis, in which studies reporting multivariable-adjusted RRs (with corresponding 95% CIs) were synthesized using the inverse-variance method under a random-effects model [3]; an RCT-only analysis, restricted to RCTs, using the same analytical approach as in the crude-event-based analysis (i.e. Mantel–Haenszel method under a random-effects model). For all analyses, between-study heterogeneity was quantified using the DerSimonian–Laird estimator (*τ*^2^), and statistical heterogeneity was assessed with the chi-squared test and *I*^2^ statistic (with *I*^2^ > 50% indicating substantial heterogeneity). Where substantial heterogeneity was observed, 95% prediction intervals (PIs) were calculated to reflect the expected dispersion of true effects across settings. Sensitivity analyses were conducted *via* the leave-one-out approach, and publication bias was evaluated using Begg’s rank correlation test and Egger’s regression test. All statistical analyses were performed using Review Manager (RevMan) version 5.3 and Stata 13.0.

## Results

### Study selection and baseline characteristics

**Figure 1.**
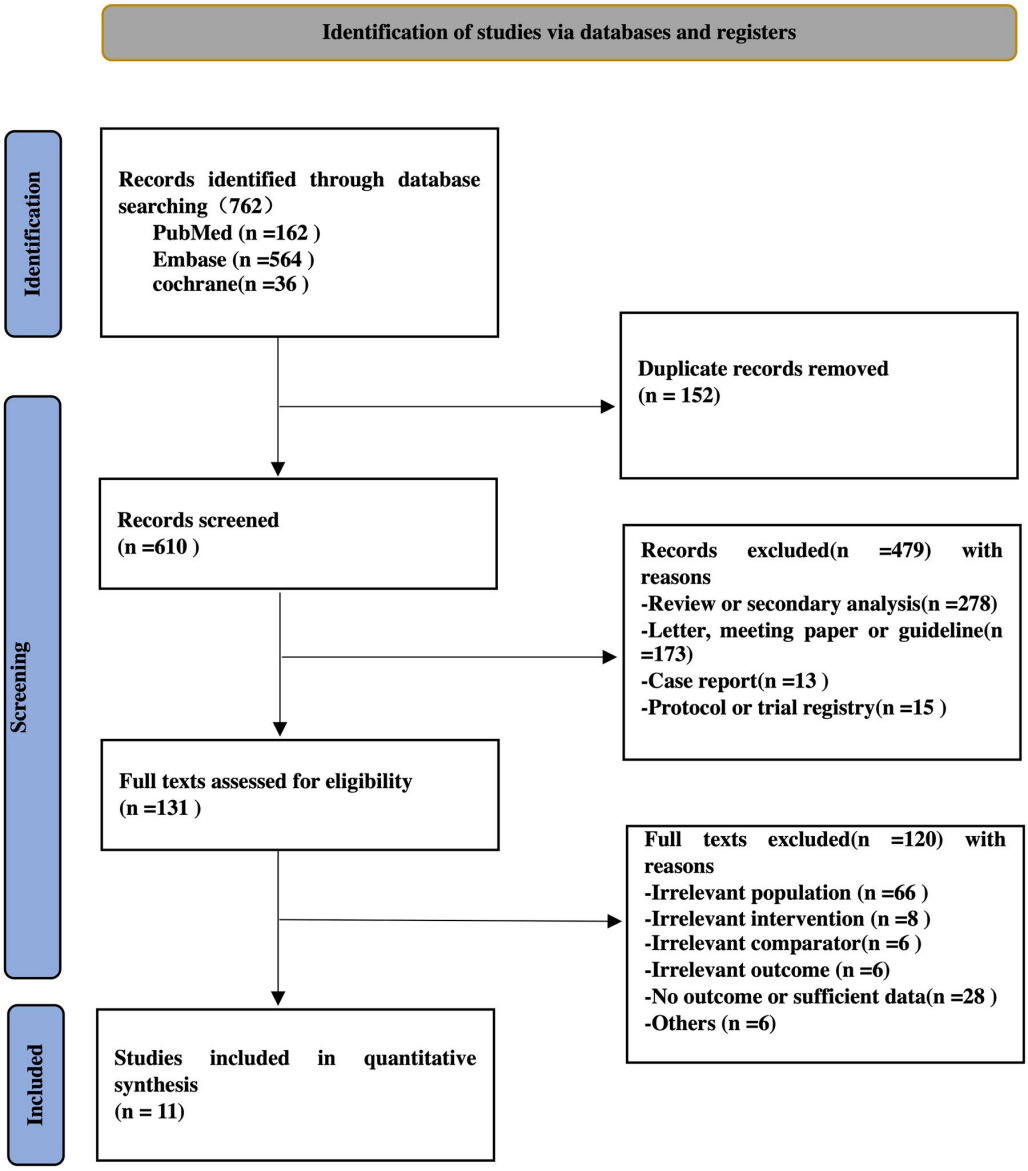
The PRISMA flow diagram for the selection of studies. PRISMA preferred reporting items for systematic reviews and meta-analyses.

A systematic search of the selected databases yielded a total of 762 results, 152 of which were duplicates. Finally, after screening the abstracts and full articles, 11 studies were identified. Of these, three RCTs (*n* = 341) and eight observational studies (*n* = 48,053) were included, comprising 48,394 patients. A flowchart detailing the inclusion and exclusion process is shown in Figure 1. The study characteristics are presented in Table 1.

**Table 1.** Characteristics of the studies included in this meta-analysis.

Author	Study design	Location	DOACs, n	Warfarin, n	Type and dose	Age mean (SD)	Sex Female. (%)	CHA2DS-VASc-score, mean	HTN, *n* (%)	DM, *n* (%)	Aspirin
Reinecke, 2023 [14]	RCT	Germany	48	49	A 2.5 mg bid	74.4 ± 18, 74.8 ± 7.9	35.4 24.5	4.5 ± 1.62 4.54 ± 1.49	NA	NA	33.3 34.7
Pokorney, 2022 [34]	RCT	USA	82	72	A mixed dose	69 ± 11.1 68 ± 8.89	41.5 30.6	4 ± 1.48 4 ± 1.48	96.3 93.1	51.2 65.3	36.7 45.7
DeVriese, 2021 [28]	RCT	Belgium	46	44	R 10 mg qd	79.9 ± 7.04 80.3 ± 9.49	23.9 43.2	4.7 ± 1.4 4.8 ± 1.5	NA	43.5 45.5	NA
Wetmore, 2022a [13]	Retrospective cohort study	USA	2382	12517	A 5 mg bid	NA	38.6 37.5	4.3 ± 1.7 4.5 ± 1.7	96.3 95.3	76.4 76.6	17.1 16.8
Wetmore, 2022b [13]	Retrospective cohort study	USA	2257	12517	A 2.5 mg bid	NA	42.7 37.5	4.7 ± 1.7 4.5 ± 1.7	97.2 95.3	79.2 76.6	18.5 16.8
Chan, 2015b [35]	Retrospective cohort study	USA	244	8064	R mixed dose	66.9 ± 12 70.6 ± 11	39.3 38.8	2.2 ± 1.0 2.4 ± 1.0	84.9 88.5	67.8 67.9	3.4 3.1
Laville, 2024a [15]	Retrospective cohort study	France	298	8471	A mixed dose	NA	NA	NA	NA	NA	NA
Laville, 2024b [15]	Retrospective cohort study	France	166	8471	R mixed dose	NA	NA	NA	NA	NA	NA
Lin, 2021 [27]	Retrospective cohort study	Taiwan	88	3185	R 10 mg qd	75 ± 9 69 ± 12	45 49	4.0 ± 1.5 3.7 ± 1.6	82 78	41 51	57 52
Moore, 2024 [36]	Retrospective cohort study	USA	53	57	A mixed dose	68.74 ± 10.28 63.37 ± 16.18	45.3 44.5	3 ± 0.74 3 ± 1.48	NA	NA	NA
Siontis, 2018a [12]	Retrospective cohort study	USA	1034	3102	A 5 mg bid	65.16 ± 10.14 68.15 ± 11.93	40.4 45.7	4.92 ± 1.71 NA	99.8 NA	NA NA	NA
Siontis, 2018b [12]	Retrospective cohort study	USA	1317	3951	A 2.5 mg bid	71.79 ± 11.65 68.15 ± 11.93	49.6 45.7	5.54 ± 1.77 NA	99.5	NA	NA
Ionescu, 2021 [37]	Retrospective cohort study	USA	144	563	A mixed dose	68.6 ± 16 67.2 ± 13.5	43.7 40.9	NA	60.4 65.1	63.9 67.3	50 50.8
Sarratt, 2017 [38]	Retrospective cohort study	USA	40	120	A mixed dose	70.9 ± 5.25 66.6 ± 5.37	50 51.7	5 ± 1.27 5 ± 0.99	82.5 80.8	55 49.2	NA NA

A = Apixaban; R = Rivaroxaban; bid = bis in die; qd = quaque die; CHA_2_DS_2_-VASc = Congestive heart failure, Hypertension, Age ≥75 [doubled], Diabetes, Stroke [doubled]-Vascular disease, Age 65–74, Sex category [female]; DM = Diabetes Mellitus; HTN = Hypertension; VKA = vitamin K antagonist.

### Assessment of the risk of bias

All RCTs were evaluated as having low risk of bias (Supplementary Fig. S1). Most of the observational studies demonstrated a low risk of bias in the classification of interventions and measurement of outcomes. However, some studies showed a moderate risk of bias in domains such as bias due to confounding and bias due to participant selection, reflecting the observational nature of many included studies. Notably, Wetmore et al. [13] and Siontis et al. [12] were rated with an overall serious risk of bias due to deviation from intended interventions and selection of the reported result. These serious biases warrant cautious interpretation of their findings (Supplementary Fig. S2).

### Effect of apixaban and rivaroxaban vs. warfarin in dialysis patients with AF

**Figure 2.**
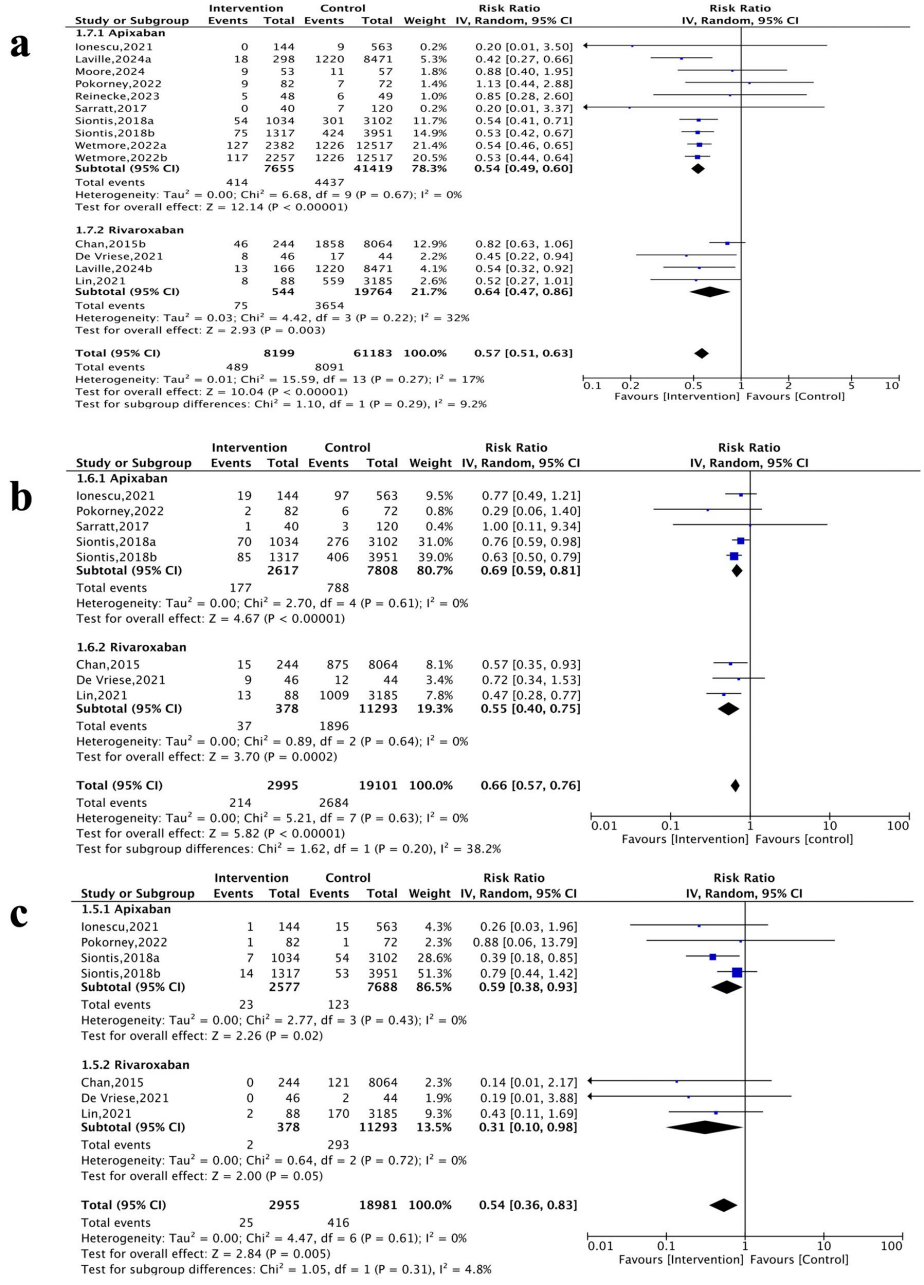
Forest plots showing risk ratio and 95% confidence interval and test for heterogeneity (*I*^2^) for safety outcomes of apixaban and rivaroxaban vs. warfarin. a) major bleeding. b) gastrointestinal bleeding. c) intracranial bleeding.

**Figure 3.**
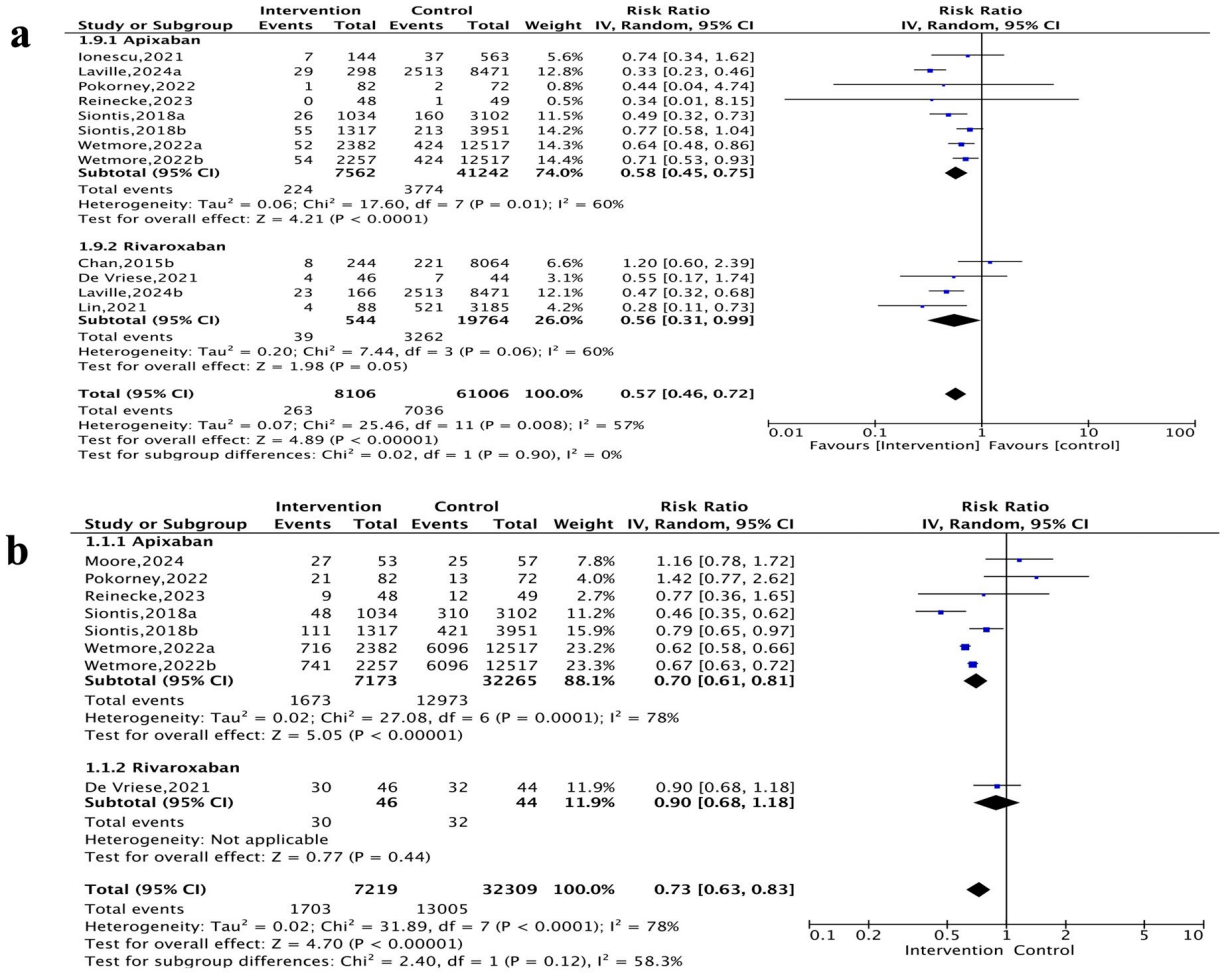
Forest plots showing risk ratio and 95% confidence interval and test for heterogeneity (*I*^2^) for efficacy endpoints of apixaban and rivaroxaban vs. warfarin. a) stroke/systemic embolism (SSE). b) all-cause mortality.

The pooled analysis showed that the safety outcomes favored apixaban and rivaroxaban, with reduced risks of major bleeding (RR 0.57, 95% CI: 0.51–0.63; *p* < 0.001; *I*^2^ = 17%, Figure 2a), gastrointestinal bleeding (RR 0.66, 95% CI: 0.57–0.76; *p* < 0.001; *I*^2^ = 0%, Figure 2b), and intracranial bleeding (RR 0.54, 95% CI: 0.36–0.83; *p* = 0.005; *I*^2^ = 0%, Figure 2c). Compared with warfarin, apixaban and rivaroxaban were associated with lower risks of SSE (RR 0.57, 95% CI: 0.46–0.72; *p* < 0.001; *I*^2^ = 57%; PI: 0.25–1.29, Figure 3a) and all-cause mortality (RR 0.73, 95% CI: 0.63–0.83; *p* < 0.001; *I*^2^ = 78%; PI: 0.47–1.14, Figure 3b), although both outcomes exhibited substantial heterogeneity.

Leave-one-out sensitivity analyses indicated that no individual study disproportionately influenced the pooled effect estimate (Supplementary Figures S3a–S7a, Figure S8). For the outcome of all-cause mortality, we performed additional sensitivity analyses to assess the impact of potentially dominant studies and to evaluate the robustness of the observed association. Exclusion of the study by Wetmore et al. (2022a) did not materially alter the results: the association between apixaban and rivaroxaban and reduced all-cause mortality remained statistically significant (RR 0.78; 95% CI: 0.64–0.96, Figure S9). Furthermore, no evidence of publication bias was detected using either Begg’s or Egger’s tests (Supplementary Figures S3b–S7b).

The pooled analysis of three RCTs showed apixaban and rivaroxaban were associated with lower risks of SSE (RR 0.50, 95% CI: 0.19–1.35; *p* = 0.17; *I*^2^ = 0%, Figure 5d) and all-cause mortality (RR 0.95, 95% CI: 0.74–1.23; *p* = 0.71; *I*^2^ = 5%, Figure 5e) compared with warfarin, although these differences did not reach statistical significance. Regarding safety, apixaban and rivaroxaban demonstrated non-significant reductions in major bleeding (RR 0.70, 95% CI: 0.39–1.25; *p* = 0.22; *I*^2^ = 20%, Figure 5a), gastrointestinal bleeding (RR 0.60, 95% CI: 0.30–1.21; *p* = 0.15; *I*^2^ = 2%, Figure 5b), and intracranial hemorrhage (RR 0.44, 95% CI: 0.06–3.34; *p* = 0.43; *I*^2^ = 0%, Figure 5c). (Supplementary Fig. 5).

### Effect of mixed-dose apixaban vs. warfarin in dialysis patients with AF

Compared with warfarin, mixed-dose apixaban was associated with a reduced risk of major (RR 0.54, 95% CI: 0.49–0.60; *p* < 0.001; *I*^2^ = 0%, Figure 2a), gastrointestinal bleeding (RR 0.69, 95% CI: 0.59–0.81; *p* < 0.001; *I*^2^ = 0%, Figure 2b), and intracranial bleeding (RR 0.59, 95% CI: 0.38–0.93; *p* = 0.02; *I*^2^ = 0%, Figure 2c). Regarding efficacy, mixed-dose apixaban was associated with lower risks of SSE (RR 0.58, 95% CI: 0.45–0.75; *p* < 0.001; *I*^2^ = 60%, PI: 0.29–1.16, Figure 3a) and all-cause mortality (RR: 0.70, 95% CI: 0.61–0.81; *p* < 0.001; *I*^2^ = 78%; PI: 0.48–1.02, Figure 3b).

### Effect of mixed-dose rivaroxaban vs. warfarin in dialysis patients with AF

Pooled results showed that mixed-dose rivaroxaban was associated with a 36% reduction in major bleeding (RR 0.64, 95% CI: 0.47–0.86; *p* = 0.003; *I*^2^ = 32%, Figure 2a), a 45% reduction in gastrointestinal bleeding (RR 0.55, 95% CI: 0.40–0.75; *p* = 0.0002; *I*^2^ = 0%, Figure 2b), and a 69% reduction in intracranial bleeding (RR 0.31, 95% CI: 0.10–0.98; *p* = 0.05; *I*^2^ = 0%, Figure 2c). Rivaroxaban was associated with lower risks of SSE (RR 0.56, 95% CI: 0.31–0.99; *p* = 0.05; *I*^2^ = 60%, PI: 0.08–3.96, Figure 3a) compared with warfarin. However, limited evidence from a single study did not demonstrate a mortality benefit (RR 0.90, 95% CI: 0.68–1.18; *p* = 0.44, Figure 3b).

### Dose-specific effects of apixaban vs. warfarin in dialysis patients with AF

#### Standard-dose apixaban (5 mg twice daily)

**Figure 4.**
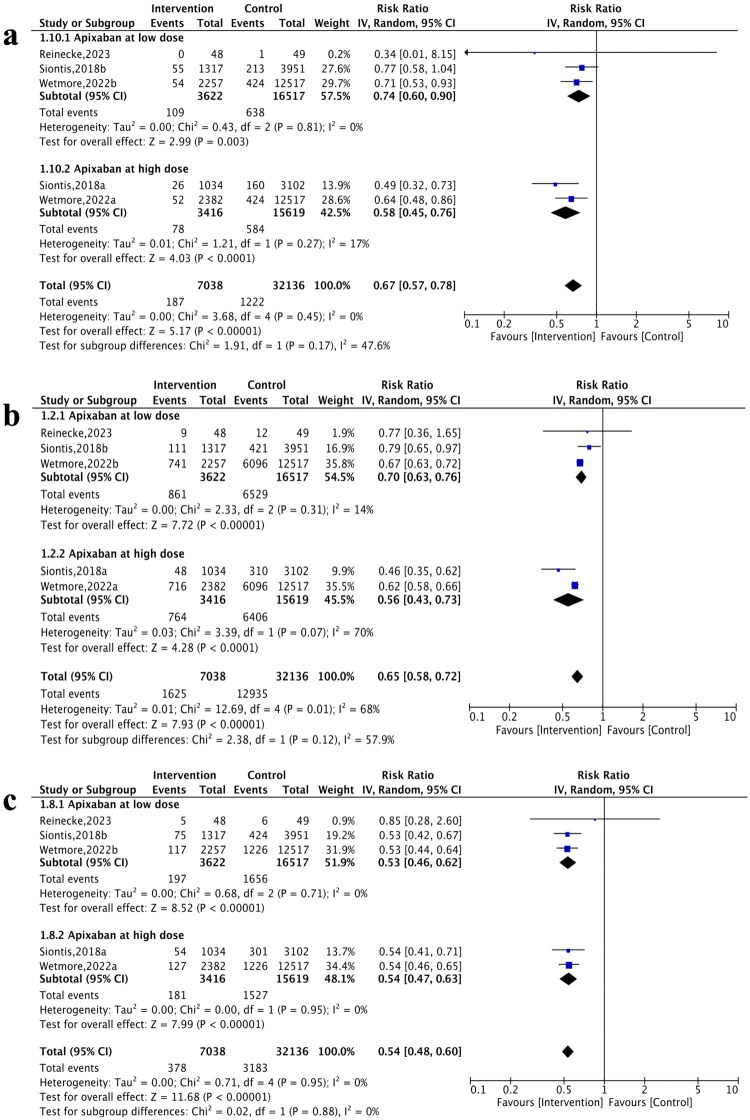
Forest plots showing risk ratio and 95% confidence interval and test for heterogeneity (*I*^2^) for primary endpoints comparing different doses of apixaban vs. warfarin a) stroke/systemic embolism (SSE). b) all-cause mortality. c) major bleeding.

Exploratory dose-specific analysis showed that standard-dose apixaban was associated with lower risks of SSE (RR 0.58, 95% CI: 0.45–0.76; *p* < 0.001; *I*^2^ = 17%, Figure 4a) and all-cause mortality (RR 0.56, 95% CI: 0.43–0.73; *p* < 0.001; *I*^2^ = 70%, PI: 0.37–0.85, Figure 4b) compared with warfarin. Regarding safety, the risk of major bleeding was reduced by 46% (RR 0.54, 95% CI: 0.47–0.63; *p* < 0.001; *I*^2^ = 0%, Figure 4c).

**Figure 5.**
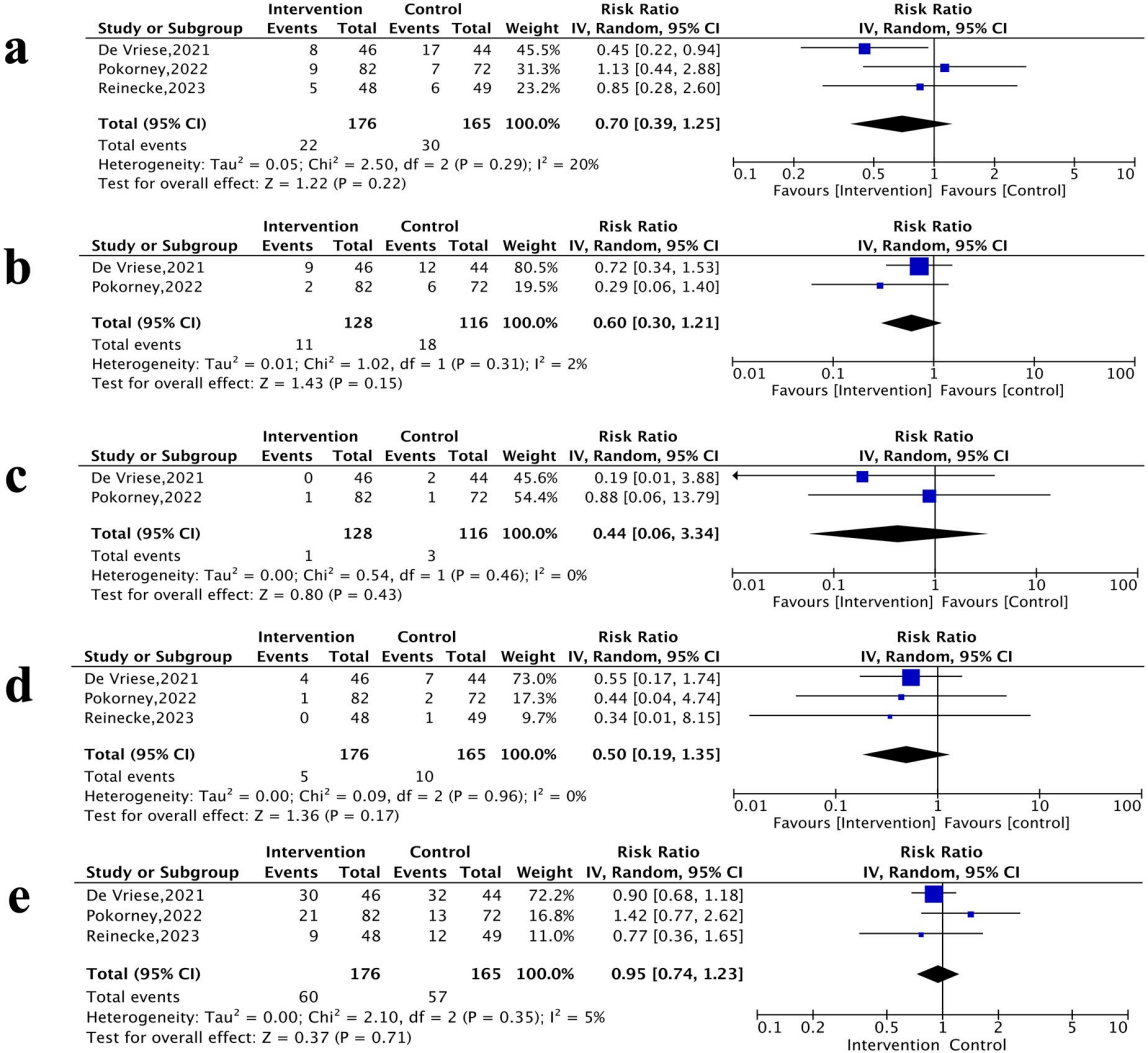
Forest plots of three RCTs showing risk ratio and 95% confidence interval and test for heterogeneity (*I*^2^) for primary endpoints comparing apixaban and rivaroxaban vs. warfarin a) major bleeding. b) gastrointestinal bleeding. c) intracranial bleeding. d) stroke/systemic embolism (SSE). e) all-cause mortality.

#### Low-dose apixaban (2.5 mg twice daily)

Exploratory dose-specific analysis showed that low-dose apixaban was associated with lower risks of SSE (RR 0.74, 95% CI: 0.60–0.90; *p* = 0.003, *I*^2^ = 0%, Figure 4a) and all-cause mortality (RR 0.70, 95% CI: 0.63–0.76; *p* < 0.001, *I*^2^ = 14%, Figure 4b). The risk of major bleeding was 47% lower (RR 0.53, 95% CI: 0.46–0.62; *p* < 0.001; *I*^2^ = 0%, Figure 4c).

## Discussion

This comprehensive meta-analysis, encompassing three RCTs and eight observational studies, provided exploratory evidence supporting the preferential use of apixaban and rivaroxaban over VKAs in dialysis-dependent patients with AF. Both standard-dose (5 mg twice daily) and low-dose (2.5 mg twice daily) apixaban, along with variable-dose rivaroxaban regimens, demonstrated s superior safety profile, with exploratory signals of improved efficacy, compared with warfarin. Nevertheless, it was critical to acknowledge the hierarchy of evidence: observational studies comprise the vast majority of studies included in this meta-analysis.

Our meta-analysis revealed that apixaban demonstrated a clinically significant safety advantage over warfarin in dialysis-dependent patients, with 46% reductions in major bleeding, 31% in gastrointestinal bleeding, and 41% in intracranial hemorrhage. These benefits were attributed to its predictable pharmacokinetics, including limited renal elimination (25% urinary excretion) and minimal dialysis clearance (4% removal during 4 h hemodialysis sessions) [19–21]. Despite these advantages, the optimal dosing strategy for apixaban remained debatable. Pharmacodynamic studies indicated that the reduced 2.5 mg twice-daily regimen in dialysis patients achieves plasma concentrations comparable to the standard 5 mg regimen in individuals with normal renal function [20]. Our meta-analysis suggested that both doses are associated with lower risks of SSE and mortality compared with warfarin, indicating that low-dose apixaban may be a viable option for patients at high bleeding risk. In contrast, a large-scale medical insurance cohort analysis reported that only standard-dose apixaban reduced t risks of thromboembolism and mortality, whereas low-dose apixaban offered no significant benefit [13]. This study strongly supported prioritizing the standard dose for its optimal benefit–risk balance. Notably, the study focused on patients who were prescribed low doses despite standard-dose labeling; therefore, its findings may not generalize to all low-dose users, especially those meeting the label criteria for low doses (e.g. age ≥80 years or weight ≤ 60 kg). The finding of another study consistent with the above adds critical insights to this controversy [22]. Its subgroup analysis suggested that the 5-mg apixaban dose confers greater clinical benefits than the 2.5-mg dose in dialysis patients, aligning with pharmacokinetic principles: dose reduction is typically reserved for drugs with fraction excreted (fe) unchanged ≥ 0.5, whereas apixaban’s low fe (0.27) implies minimal need for renal adjustment [23]. These contrasting yet complementary findings highlighted a key duality: while low-dose apixaban shows promise for selected patients at high risk of bleeding (e.g. those on concurrent antiplatelet therapy, or with advanced age, low body weight, prior major bleeding, or frailty), standard dosing remains preferable for broader populations. However, most studies in our dose-specific subgroup analyses were retrospective, which required cautious interpretation due to potential selection bias. Clinicians might prefer low-dose apixaban for older, low-weight, fragile, and more comorbid patients, potentially confounding outcomes. The absence of mortality or bleeding benefited with low-dose therapy in specific cohorts might reflect both a greater burden of comorbidity and dual therapeutic failure, with insufficient hemorrhage protection and inadequate anticoagulation. These findings underscored the urgent necessity for head-to-head RCTs, specifically designed to include high-risk populations, to ascertain optimal therapeutic dosing strategies.

While the pooled analysis suggested a favorable benefit–risk profile for apixaban and rivaroxaban compared with VKAs in terms of all-cause mortality, substantial statistical heterogeneity was observed (*I*^2^ = 78%). Our sensitivity analyses indicated that the cohort study by Wetmore et al. [13] contributed substantially to this heterogeneity. First, Wetmore et al. employed a “new-user” design, which may introduce a “healthy user bias” by excluding patients with frailty who discontinue treatment early, whereas other studies included prevalent users. Second, exposure definitions and dosing strategies varied markedly across studies. Wetmore et al. specifically isolated the “label-concordant” dosing group (apixaban 5 mg twice daily), whereas other studies pooled standard- and reduced-dose regimens. This aggregation may obscure the distinct efficacy and safety profiles of standard-dose apixaban in patients with ESRD. Third, Wetmore et al. applied inverse probability of censoring weighting (IPCW) to address differential loss to follow-up—particularly from death, a major competing risk in dialysis—whereas earlier studies lacked such rigorous adjustment. The use of IPCW likely explains the divergent mortality hazard ratios observed in this study compared with others. Fourth, Wetmore et al. used more recent data with extended follow-up, whereas other studies had shorter observation periods—further contributing to heterogeneity. Notably, despite high heterogeneity (*I*^2^ = 78%), the pooled CI for all-cause mortality remained narrow—a paradox driven by the dominance of large observational cohorts, particularly Wetmore et al. which contributed most events. Although the random-effects model accounts for between-study variance, the enormous sample size of these cohorts disproportionately influences the pooled estimate, yielding high statistical precision (narrow CI) despite substantial clinical and methodological heterogeneity. Therefore, the observed reduction in all-cause mortality should be interpreted with caution. These limitations underscore the need for validation in future, well-designed, adequately powered RCTs—particularly those stratified by age, frailty, and dosing regimen. Additionally, unmeasured treatment interruptions (e.g. bleeding events, procedural pauses) might have introduced bias, although no significant heterogeneity in bleeding outcomes was observed. Frailty-driven discontinuation patterns were prevalent in older non-dialysis AF populations. As highlighted in the EHRA consensus [24], physiological reserve depletion (e.g. sarcopenia, cognitive decline) and elevated bleeding susceptibility (e.g. fall-related injuries) frequently prompt OAC cessation despite guideline recommendations. These findings are cited here for contextual illustration and may not directly extrapolate to the dialysis population. Future trials should prioritize the standardized reporting of discontinuation patterns and tailored dosing in vulnerable subgroups.

Rivaroxaban was the second-most-studied DOAC for stroke prevention in dialysis patients. It had favorable pharmacokinetics, with 30% renal clearance and <1% hemodialysis clearance [25,26]. Dose optimization studies have shown that 10 mg daily in patients with ESRD yields therapeutic equivalence to higher doses in the general population [26], and that both low- and standard-dose regimens are comparable to warfarin in terms of thromboembolic and bleeding outcomes [27]. This finding supported its approval for use in dialysis-dependent patients with AF in Japan and Taiwan. RCT data indicated that rivaroxaban outperformed warfarin in preventing SSE, including a significant reduction in fatal and nonfatal cardiovascular events with 10 mg/day [28]. Additionally, rivaroxaban significantly reduced major bleeding (36%), intracranial hemorrhage (69%), and gastrointestinal bleeding (45%) relative to warfarin. These findings countered previous concerns regarding gastrointestinal bleeding risk and might reflect improved dose titration, proton pump inhibitor co-therapy, and the unstable INR profile of warfarin in chronic kidney disease.

The comparative advantages between apixaban and rivaroxaban remained inconclusive. A 2020 study in *the European Journal of Hematology* [29] reported no significant differences between apixaban and rivaroxaban in thromboembolic or bleeding outcomes among ESRD patients on hemodialysis, although this study was limited by non-standardized dosing. Our meta-analysis identified a non-significant trend favoring apixaban for a lower major bleeding risk, with both agents exhibiting comparable efficacy. This finding contrasts with prior network meta-analyses showing rivaroxaban’s superior thromboembolic protection at the cost of a higher bleeding risk [30]. These conflicting findings highlight the need for standardized, dose-adjusted RCTs to define agent-specific benefit–risk profiles.

Consistent with our results, prior meta-analyses have shown that both apixaban and rivaroxaban reduce the risk of gastrointestinal bleeding compared with warfarin [31]. However, these studies often pooled low-dose DOAC data without separating individual agents. Similarly, stratified RCTs and observational studies [32] were difficult to validate owing to limited randomized sample sizes, which may reflect physician hesitancy, a high dropout rate, and mortality-driven attrition. A study by Zagoridis et al. [33] reported a reduced hemorrhage risk with standard-dose apixaban (5 mg twice daily) versus warfarin in dialysis patients. However, including both AF and venous thromboembolism patients introduced heterogeneity. Notably, this study found no benefit with low-dose apixaban, in contrast to our meta-analysis, which showed a significant reduction in bleeding risk for both doses. These inconsistencies highlighted the need for large-scale, well-powered RCTs to establish clear dosing protocols. Additionally, several caveats merited attention: apixaban and rivaroxaban are not yet approved for use in dialysis populations in some countries; reimbursement barriers may limit the generalizability of our findings to certain regions; and actual prescribing patterns often reflected local regulatory or clinical practice constraints that extend beyond purely clinical evidence.

## Strengths

Overall, this meta-analysis had strengths worth noting. First, it included subgroup analyses for the dose-specific effects of apixaban, providing critically relevant insights for clinical dosing recommendations in dialysis-dependent conditions. Second, by restricting the scope to FDA-approved agents (apixaban and rivaroxaban), we reduced heterogeneity and excluded unapproved options, such as edoxaban and dabigatran. Third, an in-depth stratified analyses were conducted, which also enhanced the reliability of our results.

## Limitaions

However, several limitations must be acknowledged. First, generalizability was restricted by the inclusion of only three RCTs that focsed on dialysis-dependent patients with non-valvular AF. Second, selection and confounding biases might affect the pooled effect size owing to differences in study baseline characteristics. Third, we could not quantify anticoagulant discontinuation patterns or verify adherence to labeling criteria across cohorts. Thus, while our findings inform clinical decision-making, they do not establish definitive dosing protocols.

In summary, apixaban and rivaroxaban were associated with lower risks of bleeding—including major, gastrointestinal, and intracranial hemorrhage—compared with VKAs in dialysis-dependent patients with AF. The associations with reduced risks of SSE and all-cause mortality, although numerically favorable, were derived predominantly from observational data and exhibited substantial heterogeneity. Consistent numerical trends were observed in the RCT-only analysis, though none reached statistical significance owing to limited sample size. Similarly, exploratory findings suggesting benefits of both standard- and reduced-dose apixaban regimens in reducing the risks of SSE, mortality, and bleeding events should be interpreted as hypothesis-generating. These uncertainties underscore the need for adequately powered, dedicated randomized trials to guide anticoagulant selection and determine the optimal apixaban dosing strategy in this high-risk population.

## Supplementary Material

supplementary material.docx

## Data Availability

All data generated or analyzed during this study are included in this article and in its supplementary materials. Further inquiries can be directed to the corresponding author.
